# First principles prediction of electronic, mechanical, transport and optical properties of the silicane/Ga_2_SSe heterostructure

**DOI:** 10.1039/d2ra05723a

**Published:** 2022-11-08

**Authors:** Khang D. Pham

**Affiliations:** Institute of Applied Technology, Thu Dau Mot University Binh Duong Province Vietnam phamdinhkhang@tdmu.edu.vn

## Abstract

In this work, we investigated the electronic structure, and mechanical, transport and optical properties of the van der Waals heterostructure formed from silicane (SiH) and Janus Ga_2_SSe monolayers using first-principles prediction. The out-of-plane symmetry in the Janus Ga_2_SSe monolayer leads to the formation of two different types of Ga_2_SSe/SiH heterostructure, namely SGa_2_Se/SiH and SeGa_2_S/SiH stacking patterns. All stacking patterns of the SiH/Ga_2_SSe heterostructure are thermodynamically, mechanically and energetically stable at room temperature. Furthermore, the generation of the SiH/Ga_2_SSe heterostructure gives rise to a reduction in the band gap, demonstrating that the electrons move faster from the valence bands to the conduction bands. The SiH/Ga_2_SSe heterostructure is a semiconductor with a direct band gap of about 0.68 or 0.95 eV, depending on the stacking pattern. The SiH/Ga_2_SSe heterostructure forms type-II band alignment for all stacking patterns, indicating that the photogenerated carriers are separated effectively, thus enhancing the photocatalytic performance. Moreover, the carrier mobilities for electrons and holes of the Ga_2_SSe/SiH heterostructure are higher than those of the constituent SiH and Ga_2_SSe monolayers in both the *x* and *y* directions, suggesting that the performances of electronic devices based on the Ga_2_SSe/SiH heterostructure would be excellent and reliable. The formation of the Ga_2_SSe/SiH heterostructure also gives rise to an enhancement of the absorption coefficient in both the visible and ultraviolet regions. Our findings could give valuable guidance for the design of high-efficiency devices based on the SiH/Ga_2_SSe heterostructure.

## Abstract 2

In this work, we investigated the electronic structure, and mechanical, transport and optical properties of the van der Waals heterostructure formed from silicane (SiH) and Janus Ga_2_SSe monolayers using first-principles prediction.

## Graphical abstract

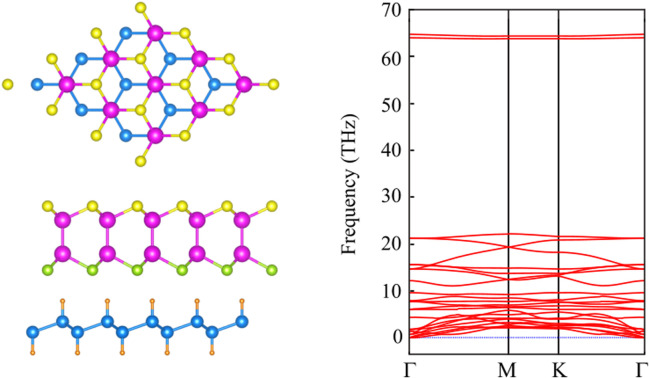

## 1 Introduction

In recent years, two-dimensional (2D) materials with extraordinary properties and wide ranging applications in different fields have become promising objects of interest in the research community, from fundamental research to practical applications. To date, a plethora of 2D materials have been successfully synthesized in experiments and theoretically predicted, such as graphene,^1^ h-BN,^2^ phosphorene^3^ and transition metal mono(di)chalcogenides (TMCs).^4,5^ These 2D materials exhibit a lot of unusual physical and chemical properties that make them promising for energy conversion and storage devices,^6^ field-effect transistors (FETs)^7^ and photocatalytic devices.^8^ For instance, the high carrier mobility in graphene^9^ makes it a promising candidate for high-speed FETs.^10^ Unlike graphene, most TMCs have moderate band gaps of about 1–3 eV that make them more attractive for high-performance electronic devices.^11,12^ However, these 2D materials have some drawbacks that may hinder their applications in various fields, including electronic, optoelectronic and photocatalytic applications. For instance, the absence of a band gap in graphene hinders its application in electronic switching devices.^13^ The mechanical instability of phosphorene in an atmospheric environment^14^ limits its use in flexible applications.^15^ Therefore, the search for novel 2D materials with unusual properties and potential applications presents many challenges for the research community.

Recently, a new type of 2D material, namely the Janus MoSSe structure has been successfully synthesized.^16,17^ The Janus MoSSe structure exhibits many unusual properties, such as Rashba spin splitting and piezoelectric polarization, that have not been found in the parent MoS_2_ and MoSe_2_ materials.^18,19^ After this success, many different Janus structures have been synthesized and predicted, including Pd_4_X_3_Y_3_ (X ≠ Y = S, Se, Te),^20^ PtSSe^21^ and MoSiGeN_4_.^22^ More recently, Guo *et al.* predicted a new Janus structure based on the parent GaS monolayer, namely the Janus Ga_2_SSe monolayer.^23^ The results demonstrated that the piezoelectric coefficients of the Janus Ga_2_SSe monolayer are enhanced up to 8.47 pm V^−1^ as compared to the GaS monolayer. The Janus Ga_2_SSe monolayer is predicted to be thermodynamically, dynamically and mechanically stable at room temperature. Additionally, a high intrinsic electron mobility of up to 10 cm^2^ V s^−1^ (ref. 24) makes the Janus Ga_2_SSe monolayer a promising candidate for ultra-small sized FETs, which can be used to replace silicon-based devices.

Silicane (SiH), a puckered honeycomb structure obtained by full hydrogenation of 2D silicene, has attracted increasing interest owing to its unique electronic characteristics and promising applications.^25,26^ The hydrogenation of 2D silicene on both edges to form the SiH material gives rise to opening of the band gap to about 3 eV,^25^ making it suitable for high-efficiency applications.^27^ Currently, in order to modify the properties of the SiH material, the research community mainly focuses on the combination of SiH and other 2D materials to create heterostructures.^28,29^ For instance, Han *et al.*^29^ performed first-principles calculations to investigate the electronic and optical properties of the combination between SiH and PtSe_2_ materials. They predicted that the combined SiH/PtSe_2_ heterostructure has enhanced optical absorption as compared to the parent SiH and PtSe_2_ materials. This enhancement suggests that the combined SiH/PtSe_2_ heterostructure can be considered as a highly efficient component for photocatalytic applications. Zeng *et al.*^27^ constructed the SiH/CeO_2_ heterostructure with enhanced visible light absorption performance compared to the constituent monolayers. They also suggested that the combined SiH/CeO_2_ heterostructure is a promising material for water splitting applications. Very recently, Lv *et al.* suggested that the UV absorptivity in the SiH/GaN heterostructure can reach 21.6%, which is greater than that of the constituent SiH and GaN monolayers. The enhancement in the UV absorptivity in the SiH/GaN heterostructure makes it suitable for ultraviolet optoelectronic devices.

In this work, we perform first-principles calculations to construct the combined SiH/Ga_2_SSe heterostructure and investigate its electronic, mechanical and optical properties. The out-of-plane symmetry in the Janus Ga_2_SSe monolayer leads to the formation of two different types of Ga_2_SSe/SiH heterostructure, namely SGa_2_Se/SiH and SeGa_2_S/SiH stacking patterns. All stacking patterns of the SiH/Ga_2_SSe heterostructure are thermodynamically, mechanically and energetically stable at room temperature. The formation of type-II band alignment in the Ga_2_SSe/SiH heterostructure is also examined. The Ga_2_SSe/SiH heterostructure has an excellent carrier mobility and a large absorption coefficient in both the visible and ultraviolet regions.

## 2 Computational methods

All calculations were performed in the framework of first-principles prediction based on density functional theory (DFT) using the Vienna *ab initio* simulation package (VASP).^30^ The Perdew–Burke–Ernzerhof (PBE) functional^31^ in the framework of the generalized gradient approximation was selected for describing the exchange–correlation energy. The geometric optimization process was fully relaxed until the forces and energy were less than 0.001 eV Å^−1^ and 10^−5^ eV, respectively. A cutoff energy of 510 eV was selected for plane-wave expansion. A Monkhorst–Pack grid with a 9 × 9 × 1 *k*-point mesh was selected for all the optimization process and electronic features calculations. A vacuum thickness of 40 Å was used to prevent the interaction between adjacent layers along the *z* direction. The weak interlayer coupling existing in most layered heterostructures was described by the DFT-D3 Grimme method.^32,33^ Additionally, the Heyd–Scuseria–Ernzerhof (HSE06) functional^34^ with the mixing parameter *α* = 0.25 was also examined to obtain more accurate band gaps of the materials. The phonon dispersion curves were calculated and *ab initio* molecular dynamics (AIMD) simulations were carried out using a 3 × 3 × 1 supercell to check the thermodynamical stability of the materials. The AIMD simulations were carried out with the PBE functional using the NVT ensemble at room temperature of 300 K. The simulations are governed by the Nosé–Hoover thermostat over 4 ps with a time step of 1 fs. The interval for saving snapshots in the trajectories is 1 ps.

The binding energy of the heterostructure is calculated as:1
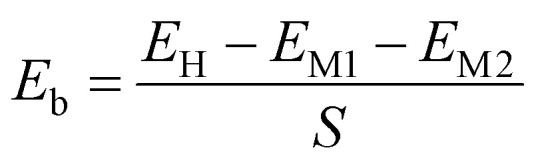
Here, the total energies of the heterostructure, and the constituent Ga_2_SSe and SiH monolayers are defined by *E*_H_, *E*_M1_ and *E*_M2_, respectively. *S* stands for the surface area of the considered heterostructure.

## 3 Results and discussion

**Fig. 1:**
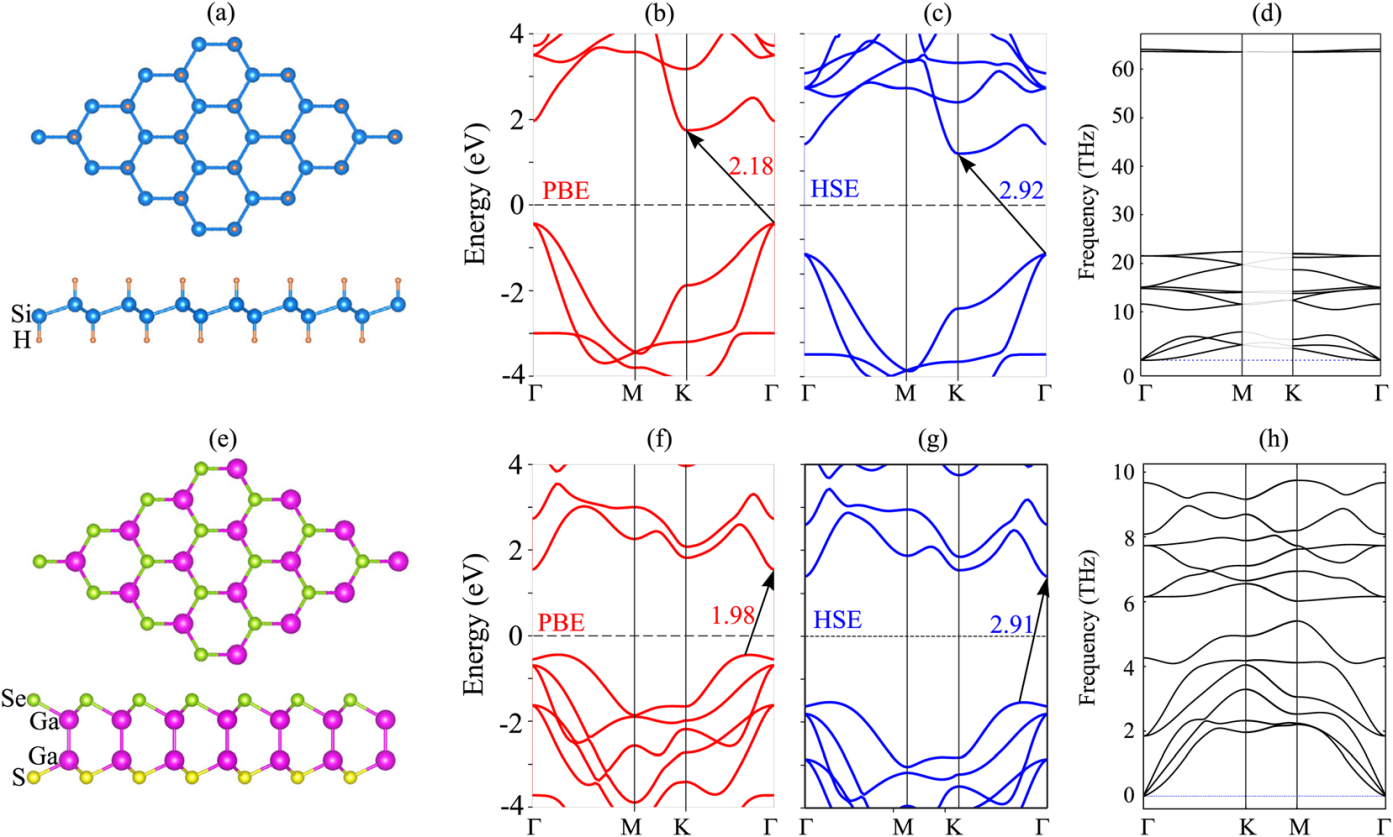
(a and e) The optimized atomic structure, (b and f) PBE band structure, (c and g) HSE band structure and (d and h) phonon spectra of (a–d) SiH and (e–h) Janus Ga_2_SSe monolayers.

We first investigate the atomic and electronic characteristics of the SiH and Janus Ga_2_SSe monolayers. The optimized atomic structures of SiH and Janus Ga_2_SSe monolayers are depicted in Fig. 1(a) and (e), respectively. The SiH monolayer shows a buckled hexagonal atomic structure, while the Janus Ga_2_SSe monolayer exhibits a layered one. The calculated lattice parameters of SiH and Ga_2_SSe monolayers after geometric optimization are 3.86 and 3.73 Å, respectively. The small difference between the lattice parameters of SiH and Ga_2_SSe monolayers gives rise to the generation of a small lattice mismatch in their heterostructure. The band structures of these monolayers given by the PBE and HSE06 methods are depicted in Fig. 1. The SiH monolayer exhibits semiconducting behavior with an indirect band gap of 2.18/2.92 eV for the PBE/HSE method. The lowest unoccupied conduction band (LUB) is located at the K point, while the highest occupied valence band (HOB) is located at the Γ point. Both the PBE and HSE methods predict the same semiconducting behavior of the SiH monolayer. Similarly, the Janus Ga_2_SSe monolayer is also a semiconductor with an indirect band gap, generated between the LUB at the Γ point and the HOB along the Γ–K path. The calculated band gap of the Janus Ga_2_SSe monolayer is 1.98/2.91 eV for the PBE/HSE method. One can find that the PBE functional can provide correct descriptions of 2D materials compared to the HSE06 functional. Therefore, we further perform all the following calculations using the PBE functional because of low computational resources. Furthermore, to verify the stability of both SiH and Ga_2_SSe monolayers, we present their phonon spectra, as shown in Fig. 1(d) and (h). It is clear that all the phononic frequencies of SiH and Ga_2_SSe monolayers are positive, verifying their dynamical stability.

**Fig. 2:**
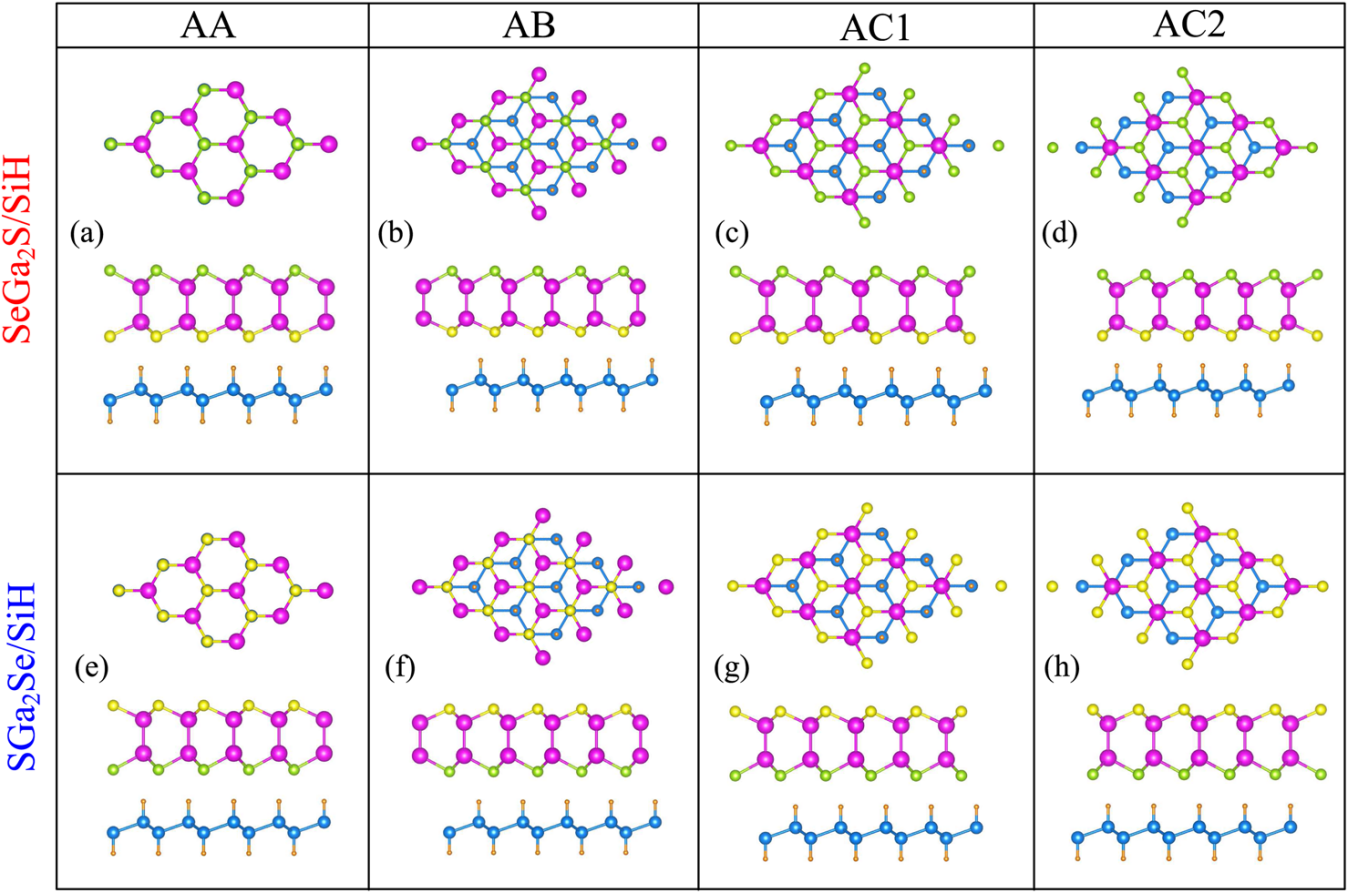
Optimized atomic structures of the SeGa_2_S/SiH heterostructure for different stacking patterns of (a) AA, (b) AB, (c) AC1 and (d) AC2 and optimized atomic structures of the SGa_2_Se/SiH heterostructure for different stacking patterns of (e) AA, (f) AB, (g) AC1 and (h) AC2.

We now design the composed heterostructure, generated from Janus Ga_2_SSe and SiH monolayers. Owing to the small difference in the lattice parameters between Janus Ga_2_SSe and SiH monolayers, to design the Ga_2_SSe/SiH heterostructure, we use a (1 × 1) unit cell of the Ga_2_SSe monolayer to match with a (1 × 1) unit cell of the SiH monolayer. The lattice parameter of the composed Ga_2_SSe/SiH heterostructure is obtained to be 3.80 Å, giving rise to a small lattice mismatch of about 1.7%. This small lattice mismatch makes the heterostructure energetically favorable and suggests that it can be synthesized easily in experiments. The out-of-plane symmetry in the Janus Ga_2_SSe monolayer leads to the formation of two different types of Ga_2_SSe/SiH heterostructure, namely SGa_2_Se/SiH and SeGa_2_S/SiH stacking patterns. Additionally, each stacking pattern of the Ga_2_SSe/SiH heterostructure includes four different stacking configurations, including AA, AB, AC1 and AC2. All stacking configurations of the Ga_2_SSe/SiH heterostructure are depicted in Fig. 2. After geometric optimization, we can obtain the interlayer separation *d* between the hydrogen layer in the SiH layer and the sulfur (selenide) layer in the Janus Ga_2_SSe layer, which is listed in Table 1. One can find that the interlayer separation of the Ga_2_SSe/SiH heterostructure for different stacking configurations is in the range from 2.22 to 2.27 Å. The AC2 stacking configuration of the SGa_2_Se/SiH heterostructure is considered to be the most energetically stable because of the shortest interlayer separation and the lowest binding energy. Furthermore, one can find that the binding energy of the Ga_2_SSe/SiH heterostructure is consistent with that of previously reported SiH-based heterostructures.^29^ Furthermore, it can be seen from Table 1 that the binding energy of the SGa_2_Se/SiH heterostructure is lower than that of the SeGa_2_S/SiH heterostructure. The reason for such a difference is that the electronegativity of Se atoms is slightly higher than that of S atoms. This finding was also observed in previous reports.^35–37^

**Table 1:** Calculated interlayer separation (*d*, Å), binding energy (*E*_b_, meV Å^−2^), band gap (*E*_g_, eV) and band alignment of the Ga_2_SSe/SiH heterostructure for different stacking configurations

Stacking configuration		*d*, Å	*E*_b_, meV Å^−2^	*E*_g_, eV	Band alignment
SeGa_2_S/SiH	AA	2.25	−10.93	0.68	Type-II
	AB	2.24	−11.19	0.70	Type-II
	AC1	2.25	−11.23	0.68	Type-II
	AC2	2.27	−10.95	0.66	Type-II
SGa_2_Se/SiH	AA	2.26	−11.65	0.93	Type-II
	AB	2.23	−11.86	0.97	Type-II
	AC1	2.25	−11.84	0.96	Type-II
	AC2	2.22	−11.95	0.92	Type-II

**Fig. 3:**
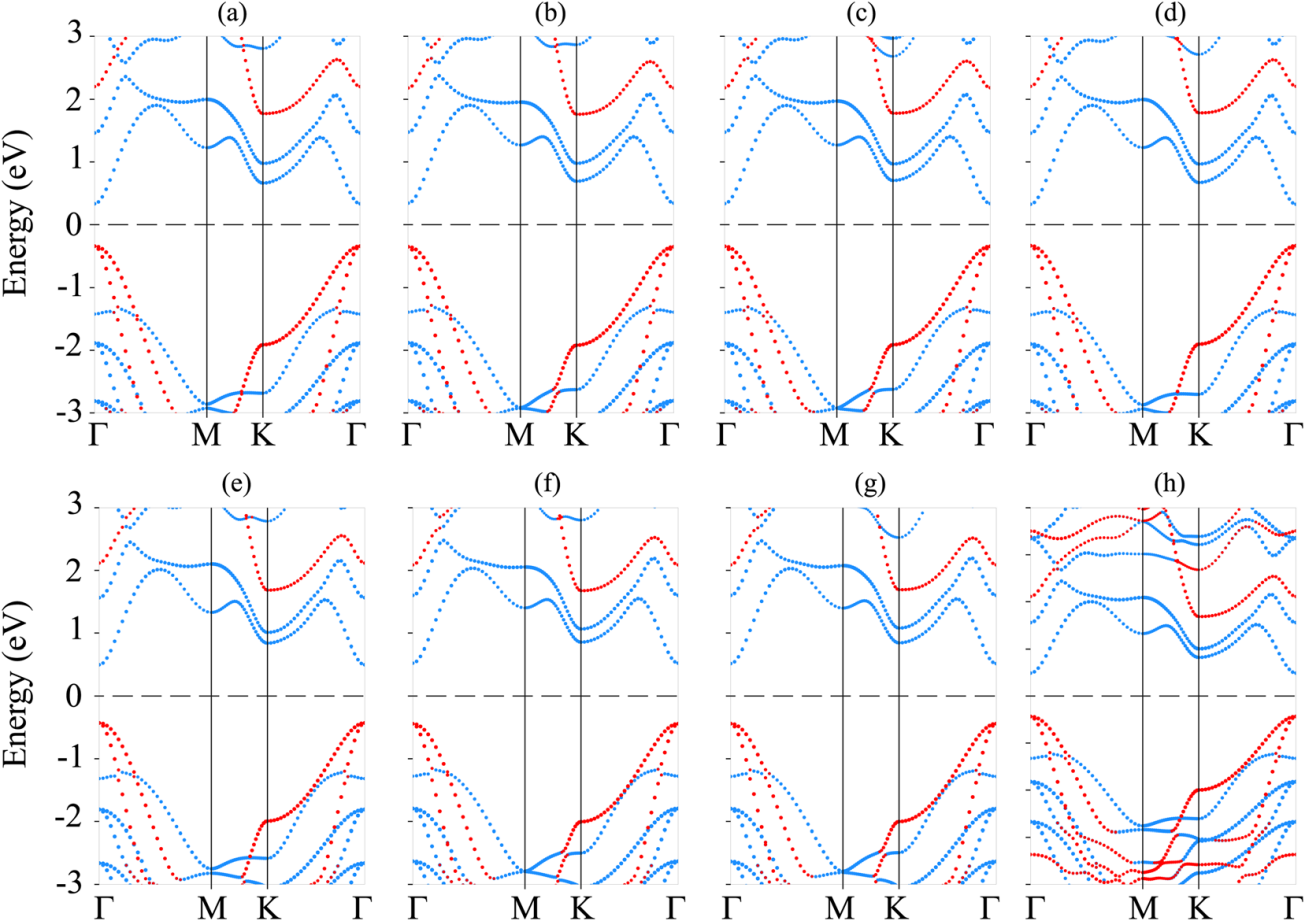
Projected band structures of (a–d) SeGa_2_S/SiH and (e–h) SGa_2_Se/SiH heterostructures for different stacking patterns of (a and e) AA, (b and f) AB, (c and g) AC1 and (d and h) AC2. The contributions of the SiH and Ga_2_SSe layers are highlighted by the red and blue lines, respectively.

The electronic band structures of the Ga_2_SSe/SiH heterostructure for different stacking configurations are depicted in Fig. 3. One can find that all stacking configurations of the Ga_2_SSe/SiH heterostructure are semiconductors with direct band gaps. Both the CBM and VBM of the Ga_2_SSe/SiH heterostructure for all stacking configurations are located at the Γ point. Thus, the generation of the Ga_2_SSe/SiH heterostructure leads to conversion from indirect semiconductors in the constituent Ga_2_SSe and SiH monolayers to a direct semiconductor. Furthermore, the band gaps of the Ga_2_SSe/SiH heterostructure are listed in Table 1. It is obvious that the band gaps of the SeGa_2_S/SiH heterostructure are smaller than those of the SGa_2_Se/SiH heterostructure. The average band gap of the SeGa_2_S/SiH heterostructure is about 0.68 eV, while it is 0.95 eV for the SGa_2_Se/SiH heterostructure. However, these values of the band gaps of the composed Ga_2_SSe/SiH heterostructure for all stacking configurations are smaller than those of the constituent Ga_2_SSe and SiH monolayers. Thus, the generation of the Ga_2_SSe/SiH heterostructure gives rise to a reduction in the band gap, demonstrating that the electrons move faster from the valence bands to the conductions bands.

Interestingly, when the Ga_2_SSe/SiH heterostructure is formed, it leads to the formation of type-I, type-II or type-III band alignment. According to the contributions of the Ga_2_SSe and SiH layers to the band structures of the Ga_2_SSe/SiH heterostructure, we find that all stacking configurations of the Ga_2_SSe/SiH heterostructure form type-II band alignment. The CBM of the Ga_2_SSe/SiH heterostructure for all considered stacking configurations is contributed by the Ga_2_SSe layer, while the VBM is dominated by the SiH layer. The formation of type-II band alignment in the Ga_2_SSe/SiH heterostructure indicates that the photogenerated carriers are separated effectively, enhancing the photocatalytic performance.

**Fig. 4:**
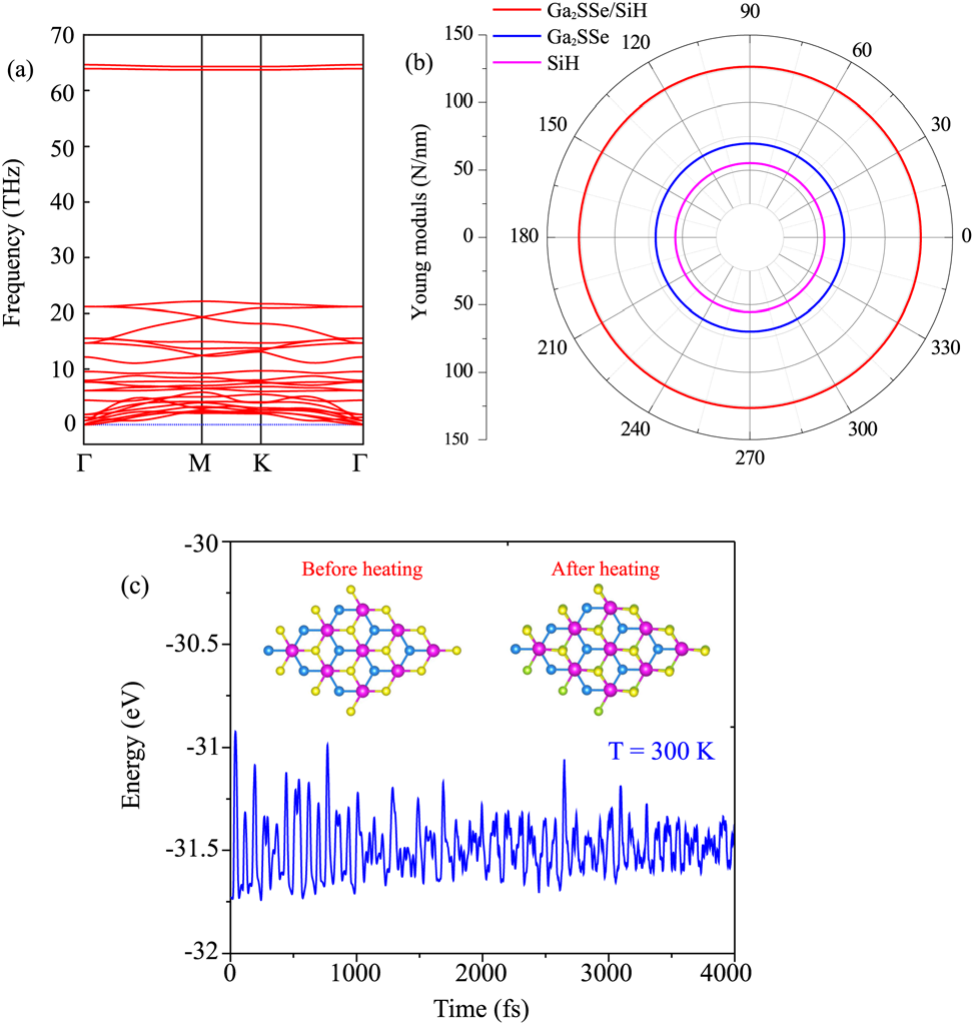
(a) Phonon spectrum, (b) Young’s modulus and (c) the total energy fluctuation as a function of time of the most energetically favorable stacking pattern of the heterostructure formed between the Janus Ga_2_SSe and SiH monolayers. Snapshots of the Ga_2_SSe/SiH heterostructure before and after heating for 4000 fs are presented in (c).

As discussed above, the AC2 stacking configuration of the SGa_2_Se/SiH heterostructure is the most energetically favorable stacking pattern; we thus focus on this pattern in all the following discussion. First, we check its dynamical, mechanical and thermodynamical stability by calculating the phonon spectrum and elastic constants, and performing *ab initio* molecular dynamics simulation, as depicted in Fig. 4. The phonon dispersion curves of the SGa_2_Se/SiH heterostructure shown in Fig. 4(a) show that all frequencies are positive, suggesting that such a heterostructure is dynamically stable in the ground state. The mechanical properties are examined to evaluate the mechanical stability of the heterostructure. The elastic constants can be obtained by calculating six different finite distortions of the lattice from the stress–strain relation as follows:^38^2
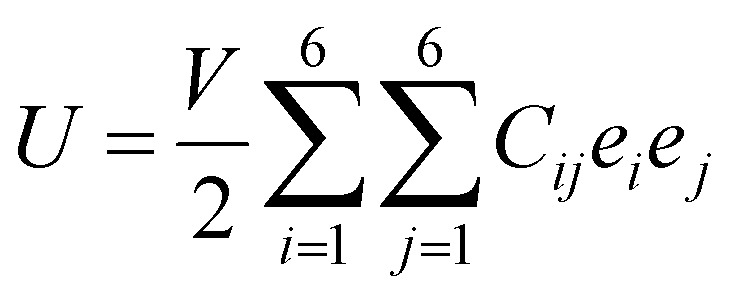
Here, the energy of the elastic potential and the volume of the undistorted lattice cell are defined by *U* and *V*, respectively. *C*_*i*,*j*_ is the matrix of the elastic constants, *i.e.* the second-order elastic constants in the Voigt notation and *e*_*i*_*e*_*j*_ is the matrix element of the strain vector. It should be noted that 1, 2 and 6 represent the *xx*, *yy* and *xy* directions of strain. The matrix of the elastic constants for 2D systems can be reduced to:
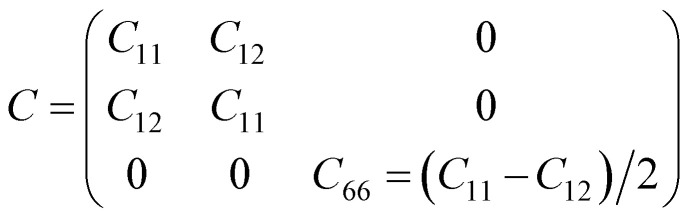


Our calculations show that there are two independent elastic constants, which are calculated to be *C*_11_ = 133.26 N m^−1^ and *C*_12_ = 30.21 N m^−1^. One can find that these values of the elastic constants of the Ga_2_SSe/SiH heterostructure meet the Born–Huang criteria, *i.e. C*_11_ > 0 and *C*_11_ − *C*_12_ > 0. This finding suggests that the Ga_2_SSe/SiH heterostructure is mechanically stable. Additionally, the Young’s modulus *Y* = (*C*_11_^2^ − *C*_12_^2^)/*C*_11_ of the Ga_2_SSe/SiH heterostructure is depicted in Fig. 4(b). The calculated Young’s moduli for the Ga_2_SSe/SiH heterostructure, and isolated Ga_2_SSe and SiH monolayers are obtained to be 126.41, 69.73 and 55.17 N m^−1^, respectively. One can find that the Young’s modulus of the Ga_2_SSe/SiH heterostructure is still greater than that of both the Ga_2_SSe and SiH monolayers, confirming satisfactory mechanical stability. The thermal stability of the Ga_2_SSe/SiH heterostructure is also evaluated by performing AIMD simulation, as shown in Fig. 4(c). It is obvious that there is a small fluctuation of the total energy and no observed structural deformation in the Ga_2_SSe/SiH heterostructure, confirming that it is thermally stable at room temperature.

**Fig. 5:**
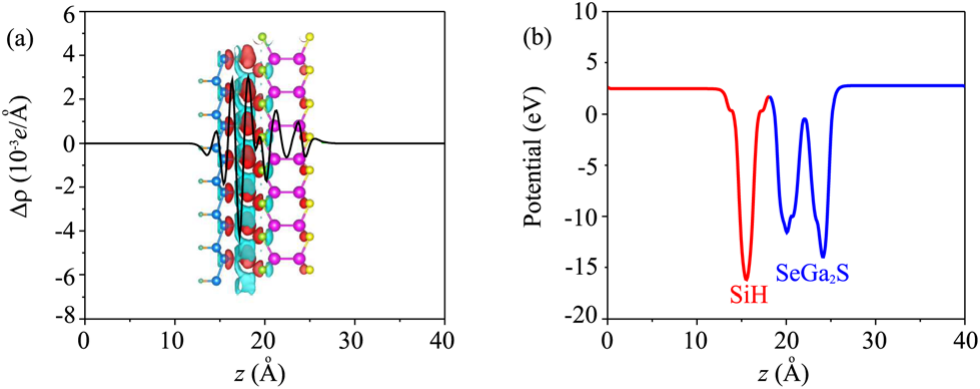
(a) Planar-averaged charge density difference and (b) electrostatic potential of the most energetically favorable stacking pattern of the heterostructure formed between Janus Ga_2_SSe and SiH monolayers. The inset in (a) represents the 3D charge density difference. Red and cyan regions represent the positive and negative charges, respectively.

Moreover, when the Ga_2_SSe/SiH heterostructure is formed, it leads to redistribution of charges. The charge redistribution of the Ga_2_SSe/SiH heterostructure can be visualized as follows:

3 Δ*ρ* = *ρ*_H_ − *ρ*_M1_ − *ρ*_M2_

where *ρ*_H_, *ρ*_M1_ and *ρ*_M2_, respectively, are the charge densities of the Ga_2_SSe/SiH heterostructure, and isolated Ga_2_SSe and SiH monolayers. Red and cyan regions in the inset of Fig. 5(a) represent the charge depletion and accumulation, respectively. It shows that the charges are depleted on the side of the SiH layer, whereas they are accumulated on the side of the Ga_2_SSe layer. This finding suggests that the charges are transferred from the SiH to the Ga_2_SSe layer in the Ga_2_SSe/SiH heterostructure. The charge redistribution between two layers in the heterostructure indicates that there will be a built-in electric field, pointing from the SiH to the Ga_2_SSe layer in the heterostructure. Such a built-in electric field inhibits the recombination rate of photocarriers. Thus, the photocarriers, namely electrons and holes, are separated into two different layers, enhancing the photocatalysis performance. The electrostatic potential of the Ga_2_SSe/SiH heterostructure is depicted in Fig. 5(b), which implies that the potential of the SiH layer is lower than that of the Ga_2_SSe layer, confirming that the charges are transferred from the SiH to the Ga_2_SSe layer.

**Fig. 6:**
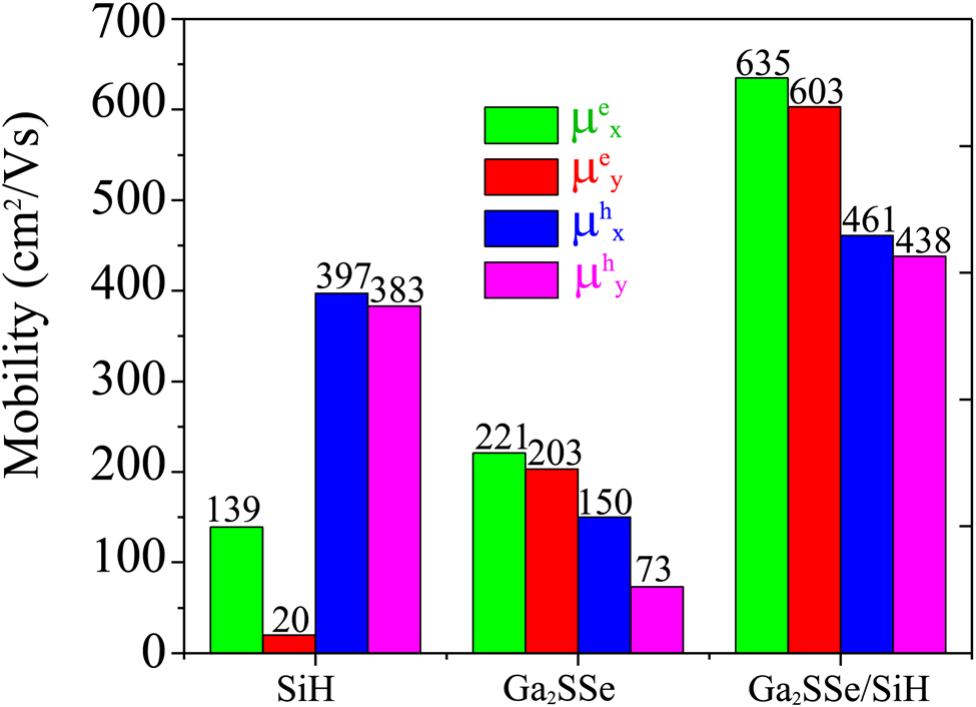
Carrier mobility for electrons and holes of SiH, Ga_2_SSe and the Ga_2_SSe/SiH heterostructure in the *x* and *y* directions.

Furthermore, for the use of the Ga_2_SSe/SiH heterostructure as a building block in high-performance electronic devices, it is important to check the transport properties, including effective mass and carrier mobility. The carrier mobility of 2D materials can be calculated as follows:4
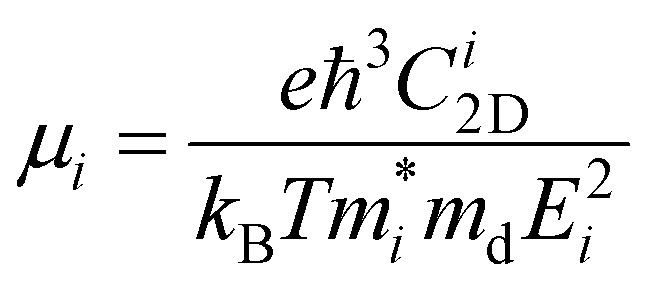
Here, *k*_B_ and ℏ are the Boltzmann constant and the reduced Planck constant, respectively. The effective mass for the *i* = *x* or *y* direction is defined by 
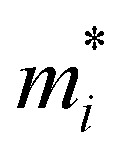
. *m*_d_ is the average of the effective masses along the *x* and *y* directions, 
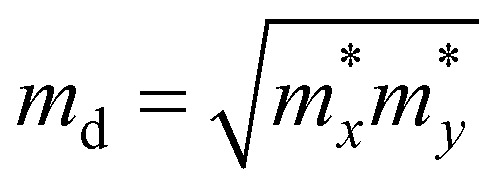
. It should be noted that the effective mass can be obtained as follows:5
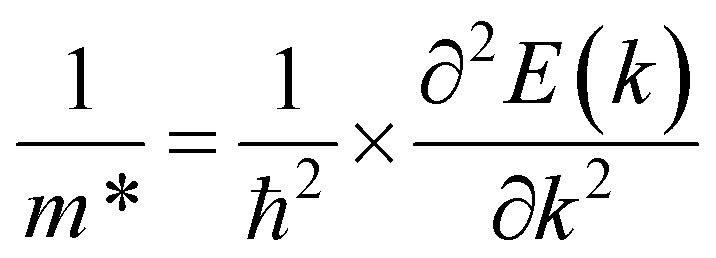
where *E*(*k*) stands for the energy with the momentum *k*. The effective mass for electrons and holes can be obtained by quadratic fitting of the band edge energy of the CBM and VBM, respectively. *E* and *C*_2D_ represent the deformation potential constant (DP) and the elastic modulus, respectively, which can be obtained as follows:6
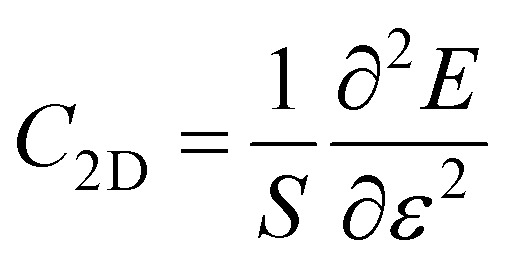
and7
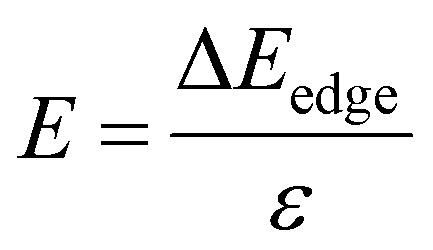
Here, *E* and *S* represent the total energy and surface area of the material, respectively. Δ*E*_edge_ is the change in the band edge of the material under small strain *ε* along the *x* or *y* direction. The calculated elastic moduli and the DP constants of the isolated SiH and Ga_2_SSe monolayers and the Ga_2_SSe/SiH heterostructure are listed in Table 2. One can find that the elastic modulus of the Ga_2_SSe/SiH heterostructure is greater than that of the constituent monolayers. More interestingly, the carrier mobilities for electrons and holes of the Ga_2_SSe/SiH heterostructure are higher than those of the constituent SiH and Ga_2_SSe monolayers in both the *x* and *y* directions, as shown in Fig. 6. The calculated carrier mobilities for electrons and holes in the *x* direction are 635 and 461 cm^2^ V^−1^ s^−1^, respectively. Whereas, they are 603 and 438 cm^2^ V^−1^ s^−1^ in the *y* direction, respectively. The electron carrier mobility of the Ga_2_SSe/SiH heterostructure is greater than its hole carrier mobility. In addition, one can find from Table 1 that the differences in the binding energies of the Ga_2_SSe/SiH heterostructure for different stacking patterns are very small. Such a finding indicates that all these patterns are energetically feasible. Therefore, the carrier mobility of the AC1 stacking configuration of the SeGa_2_S/SiH heterostructure is also calculated for comparison. We find that both the electron and hole carrier mobilities of the SeGa_2_S/SiH heterostructure are slightly smaller than those in the SGa_2_Se/SiH heterostructure. Interestingly, our findings imply that these values of carrier mobility are comparable to those in other 2D-based heterostructures,^39,40^ suggesting that the performances of electronic devices based on the Ga_2_SSe/SiH heterostructure would be excellent and reliable.

**Table 2:** Calculated elastic modulus (*C*_2D_, N m^−1^), deformation potential constant (*E*_d_, eV), effective mass (m*/*m*_0_) and carrier mobility (*μ*, cm^2^ V^−1^ s^−1^) along the *x* and *y* directions for electrons (e) and holes (h)

Materials	Carriers	*C* ^*x*^_2D_	*C* ^*y*^_2D_	*E* ^*x*^_d_	*E* ^*y*^_d_	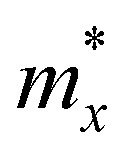	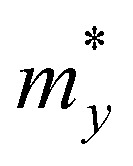	*μ*_*x*_	*μ*_*y*_
SiH	Electrons	72.54	68.99	3.728	−9.636	1.72	0.126	139	20
	Holes	72.54	68.99	−3.248	−3.224	−0.62	−0.569	397	383
Ga_2_SSe	Electrons	90.13	90.13	−10.792	−10.792	0.267	0.291	221	203
	Holes	90.13	90.13	−2.226	−2.226	−1.34	−2.77	150	73
SGa_2_Se/SiH	Electrons	176.88	167.84	−9.856	−9.856	0.25	0.138	635	603
	Holes	176.88	167.84	−5.324	−5.324	0.529	0.559	461	438
SeGa_2_S/SiH	Electrons	171.8	171.9	−9.885	−9.91	0.269	0.291	497	457
	Holes	171.8	171.9	−5.28	−5.285	−0.586	−0.535	400	437

**Fig. 7:**
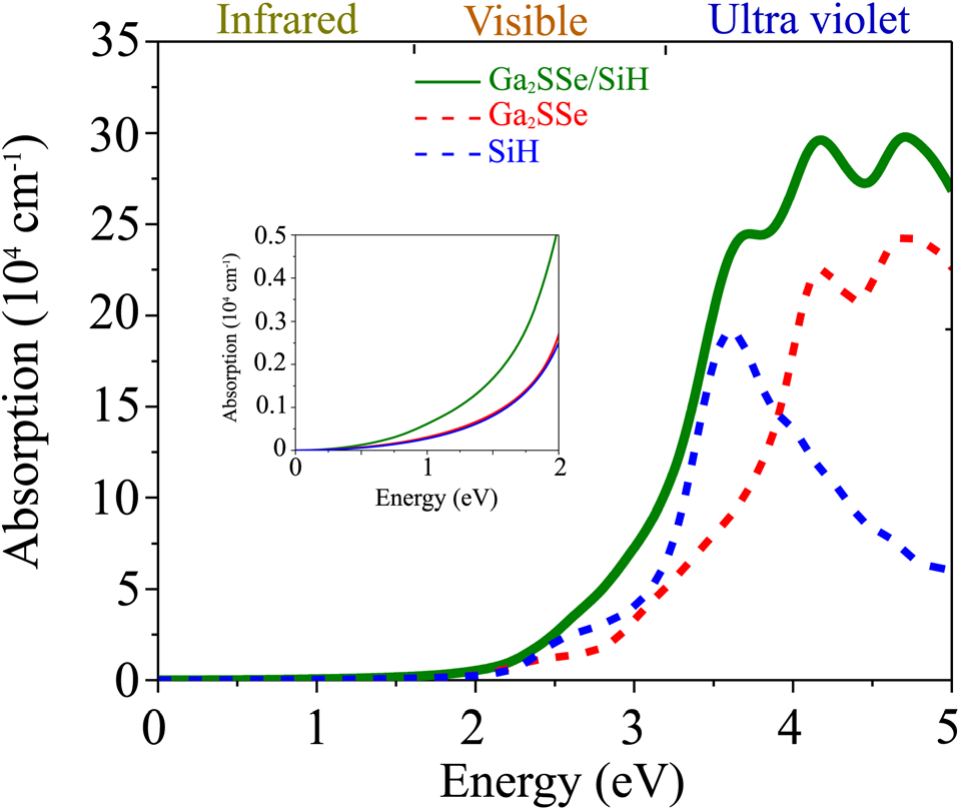
Calculated absorption coefficients of SiH and Ga_2_SSe monolayers and the Ga_2_SSe/SiH heterostructure. The inset presents the absorption coefficients of the heterostructure and the constituent monolayers in the energy range from 0 to 2 eV.

Furthermore, in view of the practical applications, it is necessary to investigate the optical properties of the heterostructure. Thus, we further calculate the optical absorption coefficient of the Ga_2_SSe/SiH heterostructure as follows:8
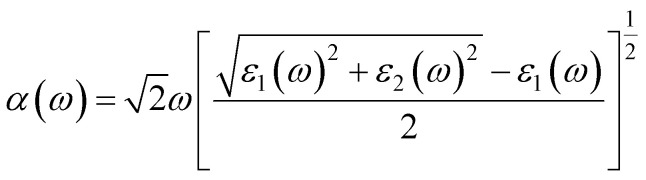
Here, *ε*_1_ and *ε*_2_ are the real and imaginary parts of the dielectric functions, respectively. *ω* stands for the photon frequency. The absorption coefficients of the SiH and Ga_2_SSe monolayers and the Ga_2_SSe/SiH heterostructure are depicted in Fig. 7. One can find that the absorption coefficient of the Ga_2_SSe/SiH heterostructure is enhanced in both the visible and ultraviolet regions as compared to the constituent SiH and Ga_2_SSe monolayers. The absorption intensity of the Ga_2_SSe/SiH heterostructure in the visible region is as high as 5 × 10^4^ cm^−1^. Whereas, the optical absorption of the Ga_2_SSe/SiH heterostructure splits into three peaks in the ultraviolet region. The maximum absorption intensity of the Ga_2_SSe/SiH heterostructure in the ultraviolet region reaches 28 × 10^4^ cm^−1^. Therefore, the formation of the Ga_2_SSe/SiH heterostructure gives rise to an enhancement of the absorption coefficient. Furthermore, the optical absorption of the Ga_2_SSe/SiH heterostructure compared to the constituent monolayers in the visible light is depicted in the inset of Fig. 7. One can find that the optical absorption of the Ga_2_SSe/SiH heterostructure is two times greater than that of the constituent SiH and Ga_2_SSe monolayers. This enhancement is related to the change in the band gap of such a heterostructure. Additionally, because of the reduction in the band gap of the Ga_2_SSe/SiH heterostructure compared to the constituent monolayers, the Ga_2_SSe/SiH heterostructure shows a good ability to absorb light in both the visible and ultraviolet regions.

## 4 Conclusions

In conclusion, we have constructed the SiH/Ga_2_SSe heterostructure with different stacking patterns using first-principles prediction. The electronic, mechanical, transport and optical properties of the most energetically favorable stacking pattern are investigated to evaluate its potential in high-efficiency applications. The SiH/Ga_2_SSe heterostructure is thermodynamically, mechanically and energetically stable at room temperature. The band gaps of the SiH/Ga_2_SSe heterostructure are smaller than those of the constituent SiH and Ga_2_SSe monolayers, suggesting that the optical absorption of such a heterostructure can be improved. The SiH/Ga_2_SSe heterostructure is a semiconductor with a direct band gap of about 0.68 or 0.95 eV, depending on the stacking pattern. The SiH/Ga_2_SSe heterostructure forms type-II band alignment for all stacking patterns, indicating that the photogenerated carriers are separated effectively, thus enhancing the photocatalytic performance. Moreover, the carrier mobilities for electrons and holes of the Ga_2_SSe/SiH heterostructure are higher than those of the constituent SiH and Ga_2_SSe monolayers in both the *x* and *y* directions, suggesting that the performances of electronic devices based on the Ga_2_SSe/SiH heterostructure would be excellent and reliable. The formation of the Ga_2_SSe/SiH heterostructure also gives rise to an enhancement of the absorption coefficient in both the visible and ultraviolet regions. Our findings could give valuable guidance for the design of high-efficiency devices based on the SiH/Ga_2_SSe heterostructure.

## Conflicts of interest

There are no conflicts to declare.

## Supplementary Material
